# Neurovascular protection in voltage‐gated proton channel Hv1 knock‐out rats after ischemic stroke: interaction with Na^+^/H^+^ exchanger‐1 antagonism

**DOI:** 10.14814/phy2.14142

**Published:** 2019-06-27

**Authors:** Weiguo Li, Rebecca Ward, Guangkuo Dong, Adviye Ergul, Paul O'Connor

**Affiliations:** ^1^ Department of Pathology & Laboratory Medicine Medical University of South Carolina Charleston South Carolina; ^2^ Ralph H. Johnson Veterans Affairs Medical Center Charleston South Carolina; ^3^ Departments of Neuroscience & Regenerative Medicine Augusta University Augusta Georgia; ^4^ Department of Physiology Augusta University Augusta Georgia

**Keywords:** Ischemic stroke, neurovascular protection, NHE inhibitor, voltage‐gated proton channel

## Abstract

Experimental studies have demonstrated protective effects of NHE‐1 inhibition on cardiac function; however, clinical trials utilizing NHE‐1 antagonists found an increase in overall mortality attributed to thromboembolic strokes. NADPH oxidase‐derived reactive oxygen species (ROS) from microglial cells have been shown to contribute to injury following stroke. We have recently demonstrated that NHE‐1 inhibition enhances ROS in macrophages in a Hv1‐dependent manner. As Hv1 protein is highly expressed in microglia, we hypothesized that “NHE‐1 inhibition may augment neurovascular injury by activating Hv1,” providing a potential mechanism for the deleterious effects of NHE‐1. The goal of this study was to determine whether neurovascular injury and functional outcomes after experimental stroke differed in wild‐type and Hv1 mutant Dahl salt‐sensitive rats treated with an NHE‐1 inhibitor. Stroke was induced using both transient and permanent of middle cerebral artery occlusion (MCAO). Animals received vehicle or NHE‐1 inhibitor KR32568 (2 mg/kg, iv) either 30 min after the start of MCAO or were pretreated (2 mg/kg, iv, day) for 3 days and then subjected to MCAO. Our data indicate that Hv1 deletion confers both neuronal and vascular protection after ischemia. In contrast to our hypothesis, inhibition of NHE‐1 provided further protection from ischemic stroke, and the beneficial effects of both pre‐ and post‐treatment with KR32568 were similar in wild‐type and Hv1^−/−^ rats. These data indicate that Hv1 activation is unlikely to be responsible for the increased incidence of cerebrovascular events observed in the heart disease patients after NHE‐1 inhibition treatment.

## Introduction

Ischemic heart disease is the leading cause of death for both men and women worldwide (Nowbar et al. 2014). The ubiquitously expressed Na^+^/H^+^ exchanger‐1 isoform (NHE‐1) mediates the 1:1 exchange of extracellular Na^+^ for intracellular H^+^ and is the primary isoform responsible for maintaining intracellular pH in cardiac myocytes (Karmazyn et al. 1999). A plethora of experimental studies have demonstrated protective effects of NHE‐1 inhibition on cardiac function in animal models (Gumina et al. 1999; Karmazyn et al. 1999; Yoshida and Karmazyn 2000; Chen et al. 2001; Karmazyn 2001; Kusumoto et al. 2001; Haist et al. 2003). Initially, these studies focused on protecting the myocardium against ischemic and reperfusion injury by preventing cellular Na^+^ overload during cellular acidification and subsequent activation of Na^+^/Ca^2+^ exchange which lead to arrhythmias. More recently, a number of studies have indicated that NHE‐1 antagonism provides long‐term protection against the development of cardiac hypertrophy, a risk factor for ischemic heart disease (Karmazyn 2001). The EXPEDITION study (5761 patients) was designed to determine whether NHE‐1 inhibition prevented coronary events in high‐risk patients (Mentzer et al. 2008). This study found a highly significant risk reduction for death or nonfatal myocardial infarction in patients treated with the selective NHE‐1 antagonist cariporide. While NHE‐1 inhibition was shown to be protective against myocardial infarction, overall mortality increased with NHE‐1 inhibitor use due to an increased incidence of cerebrovascular events attributed to thromboembolic strokes (Mentzer et al. 2008). Despite the enormous potential of NHE antagonists as cardioprotective agents, these serious side effects have precluded further development of NHE‐1 antagonists for treatment of heart disease and called for preclinical studies to identify potential mechanisms underlying the increased cerebrovascular events in patients treated with these compounds.

We have recently demonstrated that the voltage‐gated proton channel Hv1 is required for the generation of reactive oxygen species (ROS) in renal tubular cells in response to cellular acidification and that Hv1 contributes to the development of hypertension and renal injury in Dahl salt‐sensitive (SS) rats (Jin et al. 2014). Hv1 channels play multiple roles in different cells. One of the most thoroughly studied functions is the role of Hv1 in charge compensation and pH regulation during the respiratory burst in the innate immune system (DeCoursey 2013). We showed that low intracellular Na^+^ levels enhance ROS formation by NADPH oxidase in peritoneal macrophages and inhibition or genetic deletion of Hv1 prevents this response (Jin et al. 2014). Interestingly, Hv1 protein is highly expressed in the microglia, the primary resident immune cells, but not other cells in the brain (Wu et al. 2012). Like macrophages, microglia are phagocytic cells that express high levels of Hv1 (Capasso et al. 2010; DeCoursey and Hosler 2013). Recent studies have reported that mice lacking Hv1 exhibit smaller brain infarcts and better stroke outcomes by dampening NADPH oxidase‐dependent ROS generation after ischemic stroke (Wu et al. 2012). In light of these studies, we hypothesized that NHE‐1 inhibition augments neurovascular injury after stroke by activating Hv1, providing a potential mechanism for the deleterious effects of NHE‐1 inhibition in regard to cerebrovascular events. A corollary to this hypothesis is that poor stroke outcomes mediated by NHE‐1 inhibition are attenuated in the absence of Hv1. To test our hypothesis, we investigated neurovascular injury and functional outcomes in wild‐type (WT) and (Hv1^−/−^) Dahl salt‐sensitive (SS) rats treated with the potent and specific NHE‐1 inhibitor KR32568.

## Methods

### Animals

Since our past studies that showed the involvement of Hv1 in low intracellular Na^+^ or NHE‐1‐mediated ROS generation was conducted in male rats, the current study also used male WT Dahl SS rats weighing 250–350 g maintained *ad libitum* on water and a standard pellet diet containing 0.4% NaCl since weaning (Dyets, Bethelem, PA). Hv1^−/−^ SS rats were generated with zinc‐finger nuclease technology. Genomic DNA of homozygous Hv1 mutant (Hv1^−/−^) rats was sequenced to reveal an 8‐bp deletion, resulting in a frame‐shift mutation and predicted loss of full‐length HVCN1 protein. We have previously confirmed complete loss of Hv1 channel activity in this strain (Jin et al. 2014). Similar to reports from Hv1^−/−^ mice (Wu et al. 2012; Sasaki et al. 2013), these rats appear phenotypically normal and blood pressure responses are similar to those observed in the parent WT Dahl SS rat strain (Jin et al. 2014; Ray et al. 2018) (Table 1 and Fig. 3). All of the protocols were approved by the Institutional Animal Care Committee at Augusta University.

**Table 1:** Physiological parameters of animals in each group. In Experiment 1, the animals were grouped according the surgery types, which are permanent MCAO or transient MCAO. In experiments 2 and 3, the animals were grouped according to the NHE‐1 inhibitor treatment plan

Experiment 1				
Surgery	Permanent		Transient	
Animal	WT (*n* = 4)	Hv1^−/−^ (*n* = 4)	WT (*n* = 5)	Hv1^−/−^ (*n* = 3)
Age (wk)	10.5 ± 0.1	10.5 ± 0.1	10.8 ± 0.4	10.3 ± 0.4
BW (g)	252.6 ± 6.0	269.1 ± 5.3	277.2 ± 5.2	259.1 ± 10.1
BG (mg/dL)	82.3 ± 3.3	75.5 ± 3.5	86.2 ± 1.5	82.7 ± 3.3

Experiments 2 and 3						
Treatment	Vehicle		Acute		Chronic	
Animal	WT (*n* = 6)	Hv1^−/−^ (*n* = 4)	WT (*n* = 4)	Hv1^−/−^ (*n* = 5)	WT (*n* = 5)	Hv1^−/−^ (*n* = 5)
Age (wk)	11.7 ± 0.2	12.5 ± 0.1	12.1 ± 0.4	12.5 ± 0.1	12.1 ± 0.00	12.2 ± 0.1
BW (g)	330.4 ± 7.2	320.6 ± 13.0	335.7 ± 11.9	321.8 ± 9.9	322.0 ± 6.9	310.0 ± 4.8
BG (mg/dL)	77.8 ± 2.4	78.8 ± 3.9	79.8 ± 4.5	77.4 ± 3.2	78.0 ± 2.5	84.8 ± 3.1

WT, wild‐type; BW, body weight; BG, blood glucose.

### General experimental design

In order to determine the role of Hv1 in stroke injury in SS rats, multiple models of middle cerebral artery occlusion (MCAO) were used to mimic ischemic (permanent MCAO) and ischemia/reperfusion (transient suture and embolic MCAO) injury. In Experiment 1, age and weight‐matched WT and Hv1^−/−^ rats (in house colony) were randomly assigned to permanent or transient MCAO by a suture insertion (*n* = 3–5/group). In the permanent group, the suture was not removed, whereas in the transient group, the suture was pulled back after 3 h to achieve reperfusion. After assessment of neurological deficits by a composite score and grip strength test, animals were sacrificed at 24 h time point for evaluation of neuronal (infarct size) and vascular (edema, hemorrhagic transformation [HT] index, and excess brain hemoglobin [Hb]) injury. In Experiment 2, age and weight‐matched WT and Hv1^−/−^ rats were subjected to embolic MCAO and randomly assigned to vehicle or acute NHE‐1 inhibitor KR32568 (2 mg/kg, iv) treatment 30 min after the start of MCAO (*n* = 4–5/group, acute in Table 1). In Experiment 3, an additional cohort of WT and Hv1^−/−^ rats were pretreated with NHE‐1 inhibitor KR32568 (2 mg/kg/day, iv) for 3 days and then subjected to embolic MCAO (*n* = 5/group, chronic in Table 1). After assessment of sensorimotor deficits, animals were sacrificed at 72 h poststroke for evaluation of neuronal (infarct size) and vascular (edema, HT index, and excess brain Hb) injury.

### Stroke surgery

Animals were anesthetized with 5% isoflurane, and anesthesia was maintained with 2% isoflurane in 70% N_2_ and 30% O_2_ using a face mask. In the suture model, a filament was inserted up to the origin of MCA through the stump of external carotid artery (ECA). The filament was left in and pulled back from the vessel to reestablish blood flow at various time points as mentioned above. In the embolic model, a modified PTFE 160 catheter containing the clot was inserted through the ECA stump as in the suture model, and the clot was gently injected with 100 *μ*L of the sterile saline. The catheter was removed immediately after embolization. Laser Doppler imaging with a scanning laser Doppler (PIM3, Perimed; North Royalton, OH) was used to confirm successful occlusion and ensure similar levels of blood flow reduction in all groups.

### Infarct size, edema ratio, and HT analysis

The infarct size was measured after 2,3,5‐triphenyltetrazolium chloride (TTC) staining as previously described (Ergul et al. 2007). Edema was calculated as a percent (%) increase in the ischemic hemisphere versus the contralateral hemisphere, and the edema ratio was reported after normalizing for infarct size. After staining, the hemispheres were separated and deep frozen for tissue Hb quantification with QuantiChrom kit (BioAssay Systems, Hayward, CA) (Qin et al. 2007) and reported as excess Hb (mg/g protein) in the ischemic hemisphere (ischemic hemisphere minus contralateral hemisphere). A blinded investigator scored macroscopic HT index in slices B–E using a four‐point rubric (Kelly‐Cobbs et al. 2013) (0 – normal ischemic damage or hemorrhage; 1 – dispersed individual petechiae; 2 – confluent petechiae; 3 – small diffuse hemorrhage or hematoma; 4 – large diffuse hemorrhage or hematoma), and the total score for each animal was reported.

### Behavioral measurements

Neurobehavioral tests were assessed, recorded, and scored in a blinded fashion as described before (Prakash et al. 2013). Animals were handled for 5–7 days prior to behavior testing in rooms where behavior testing was to be carried out. In Experiment 1, tests included Bederson's score and grip strength tests before and after stroke at 24 prior to sacrifice. In experiments 2 and 3, tests included Bederson's score, beam walk, and adhesive removal test (ART) for evaluation of fine motor skills. Bederson's score for each rat was obtained by using four parameters which include (a) observation of spontaneous ipsilateral circling (2 for no circling, 1 for partial circling, and 0 for continuous circling), (b) contralateral hindlimb, (c) forelimb retraction which measures the ability of the animal to replace the limb after it is displaced laterally by 2 to 3 cm (2 for immediate replacement, 1 for replacement after minutes, and 0 for no replacement), and (d) resistance to push (1 or 0, depending on whether the animal is able to resist pushing or not). Maximum score of 7 was allotted to a normal rat. Beam walk ability graded based on 7‐point scale method as described by Feeney et al. (1982) and Kelly‐Cobbs et al. (2013). Composite neurological score was reported as the sum of the Bederson's score and the beam walking score on a 0–14 scale with a maximum score of 7 (Experiment 1) and 14 (experiments 2 and 3) indicating no deficits. Forelimb grip strength was measured with a standard grip strength meter (Columbus Instrument, Columbus, OH) (Terry et al. 2007). ART was examined as described previously (Li et al. 2017). Briefly, animals were trained to remove an adhesive paper dot for 5 days prior to baseline recording. Contact and removal latency were reported at baseline, Day 1, and Day 3 after MCAO. For each time point, three trials were averaged with the maximum removal latency at 180 seconds per trial.

### Effect of chronic NHE inhibition on sodium excretion and blood pressure in Dahl SS rats

Experiments were performed on 10‐week‐old WT Dahl SS rats maintained on purified AIN‐76A rodent diet (Dyets) containing 0.4% NaCl. On the day of surgery, rats were deeply anesthetized with a mixture of ketamine (40 mg/kg, im), xylazine (8 mg/kg, im), and acepromazine (4 mg/kg, im), with supplemental anesthesia administered as needed. With the use of aseptic techniques, polyvinyl catheters were placed in the femoral artery and vein for measurement of mean arterial pressure (MAP) and infusion of fluids. Catheters were tunneled subcutaneously and exteriorized at the back of the neck via a spring. Animals were maintained on warming trays during and following surgery. Analgesics and antibiotics were administered after surgery to control pain and infection. Rats were allowed to recover for 7 days before the experimental protocol started.

After recovery, MAP was recorded 24 h daily using an online data collection and analysis system (Cowley et al. 2000). Rats were housed in metabolic cages, provided water and food *ad libitum,* and 24‐h urine collected for measurement of urine albumin, protein, Na^+^, and K^+^. A vehicle infusion containing 1% DMSO in saline was administered at a rate of 6.9 *μ*L/min (iv) throughout the study. Arterial catheters were maintained patent by chronic infusion of heparinized saline (30 U/mL) at 100 *μ*L/h and daily flushing with 0.1–0.2 mL of saline. At the beginning of the experimental protocol, rats received only vehicle infusions. Following 3 days of baseline measurements, rats were switched to an 8% high NaCl diet (Dyets). At Day 3 of high NaCl diet, animals were either maintained on vehicle infusion or the vehicle switched to KR32568 (2 mg/kg/day) for the remainder of the study.

### Urine and plasma electrolytes

Samples were measured by flame photometry (IL‐943; Instrumentation Laboratories, Lexington, MA). Microalbuminuria was quantified with an Albumin Blue 580 (Molecular Probes) fluorescence assay. Proteinuria was quantified with Weichselbaum's biuret reagent on an ACE autoanalyzer (Alfa Wassermann).

### Data and statistical analysis

All data are expressed as mean ± SEM. Responses were compared using GraphPad Prism software (GraphPad). Neurovascular injury and functional outcomes after transient or permanent stroke (Experiment 1) in WT and Hv1^−/−^ rats were compared using Student's *t*‐test. For experiments with treatments, multicomparisons were made using either two‐way ANOVA and Newman–Keuls post hoc test or two‐way repeated‐measures ANOVA. The level required to reach significance was *P* < 0.05.

## Results

### Deletion of Hv1 channel confers neuronal protection after ischemic stroke

**Figure 1:**
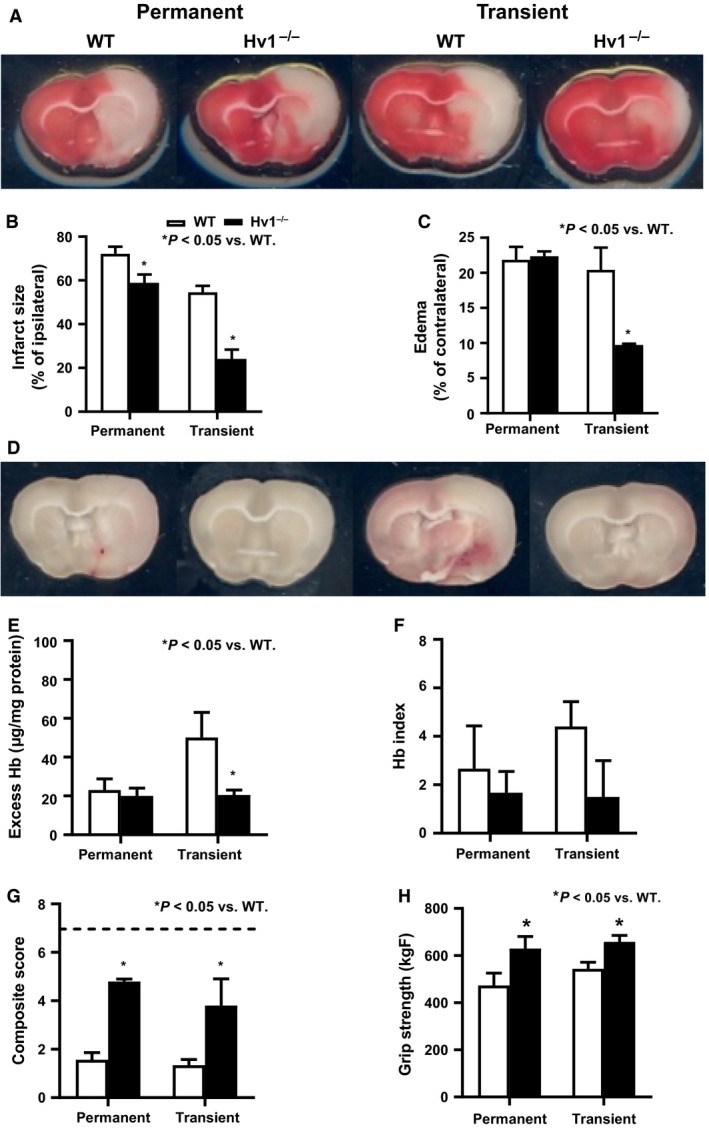
Neurovascular injury is less in Hv1^−/−^ rats after permanent or transient cerebral ischemia. Infarct size, edema, excess Hb, and HT index in the ischemic hemisphere and neurological deficits were measured 24 h after permanent or transient (3‐h occlusion/21‐h reperfusion) MCAO. (A and B) Collective analysis of TTC‐stained sections (representative images are shown on top) showed that Hv1^−/−^ rats developed smaller infarcts regardless of the stroke model. (C) On the other hand, edema was similar between two groups after permanent MCAO but significantly less in Hv1^−/−^ rats after transient MCAO. (D to F) Secondary bleeding into the brain measured by quantitative assessment of excess Hb in the ischemic hemisphere (E) or scoring of macroscopic bleeding (HT index in panel F) in the coronal sections shown on top (D) showed that ischemia reperfusion injury induced by transient MCAO increased vascular damage and Hv1^−/−^ rats were protected from this injury. (G and H) Neurological deficit was measured by 7‐point composite score and grip strength. Higher score indicates favorable outcome and normal behavior score is indicated by dashed line (*n* = 3–5/group in these studies, Student's *t*‐test comparison of WT versus Hv1^−/−^ rats in permanent or transient MCAO, significance *P* < 0.05).

Ischemic injury induced by permanent MCAO causes extensive damage indicated by infarction that covers almost entire hemisphere, which was reduced by about 2% in Hv1^−/−^ rats (Fig. 1A and B). The infarct size caused by ischemia/reperfusion injury after a transient 3 h MCAO is relatively smaller compared to permanent occlusion and Hv1 deletion reduces the infarct size more than 50%, suggesting a greater role of this channel in reperfusion injury.

### Deletion of Hv1 channel confers vascular protection after ischemia reperfusion injury

There was no difference in indices of vascular injury (edema, HT index, and Hb excess) between WT and Hv1^−/−^ rats after permanent MCAO (Fig. 1C–F). On the other hand, both edema and excess Hb in the ischemic hemisphere were significantly lower in Hv1^−/−^ rats after reperfusion injury induced by transient MCAO (Fig 1C–F).

### Deletion of Hv1 channel reduces acute neurological deficits after ischemic stroke

Sensorimotor deficits caused by permanent and transient stroke were greater in the WT animals compared to Hv1^−/−^ rats as indicated by lower composite scores and grip strength (Fig. 1G and H).

### NHE‐1 inhibition started before or after embolic stroke provides similar degree of neurovascular protection in WT and Hv1^−/−^ rats

**Figure 2:**
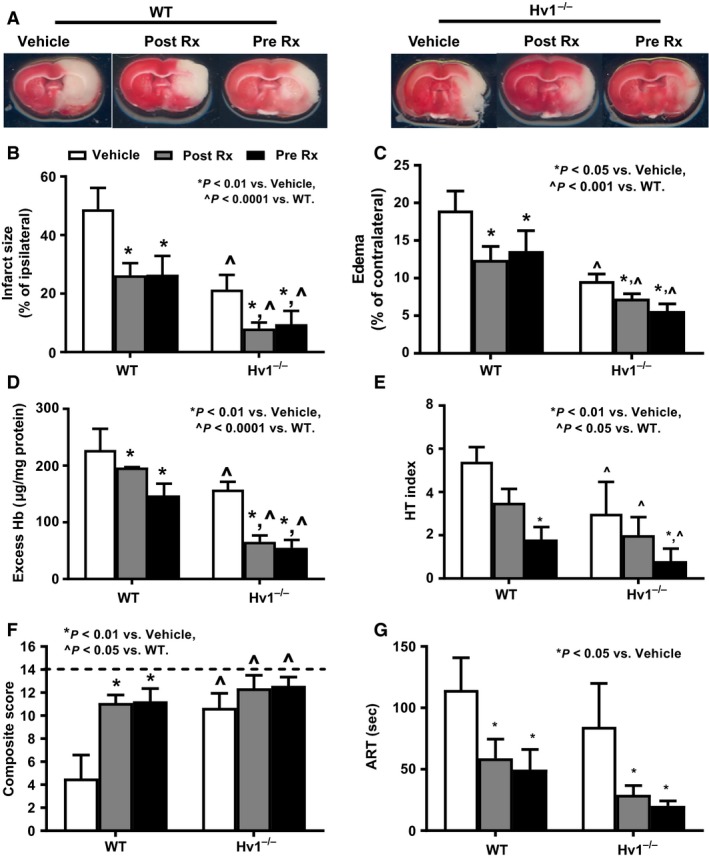
Neuroprotective effects of NHE‐1 inhibition started before or after stroke are similar in WT and Hv1^−/−^ rats. Representative brain sections with TTC staining for the infarct and edema analyses are shown on panel A. There is both a treatment and gene effect. Treatment reduces infarction (B), edema (C), excess Hb (D), and HT index (E), in both strains. All these indices of neurovascular injury are significantly lower in Hv1^−/−^ rats compared to WT. Motor deficits detected by composite score (F) at Day 3 were less in vehicle‐treated Hv1^−/−^ rats compared to WT rats. Neurological deficit was measured by 14‐point composite score. Higher score indicates favorable outcome and normal behavior score is indicated by dashed line. In the vehicle‐treated groups, both WT and Hv1^−/−^ rats showed significant impairment at Day 3 as indicated by longer ART time (G), while either pre‐ or poststroke treatment decreased the time to remove the adhesive tape (*n* = 5–6/group in these studies, two‐way ANOVA comparisons for treatment and gene effect, significance *P* < 0.05).

Infarct size measured 3 days after ischemic injury induced by embolic MCAO was smaller in Hv1^−/−^ rats compared to WT. The degree of protection was similar to that observed with transient stroke induced by suture occlusion of MCAO. One time acute treatment with NHE‐1 inhibitor KR‐32568 in the poststroke period or 3‐day pretreatment reduced infarct size by 50% in both WT and Hv1^−/−^ rats (Fig. 2A and B). Edema was less in Hv1^−/−^ rats, and both treatments had a similar effect in both groups (Fig. 2C). Excess Hb and HT score analyses (Fig. 2D and E) showed that treatment reduced bleeding in both groups and overall Hv1^−/−^ rats had lower bleeding.

### NHE‐1 inhibition improves functional outcomes

Neurological deficits after embolic stroke were measured by two different tests including composite score and ART (Fig. 2F and G). Motor deficits detected by composite score at Day 3 were less in vehicle‐treated Hv1^−/−^ rats compared to WT rats. Hence, treatment effect size was smaller compared to WT animals. ART based on the time to remove adhesive tape measures fine sensorimotor function. In the vehicle treatment groups, both WT and Hv1^−/−^ rats showed significant impairment by Day 3. Either pre‐ or poststroke treatment decreased the time to remove the adhesive tape.

### Chronic NHE inhibition does not affect blood pressure or Na^+^ excretion but enhances hypertensive proteinuria

**Figure 3:**
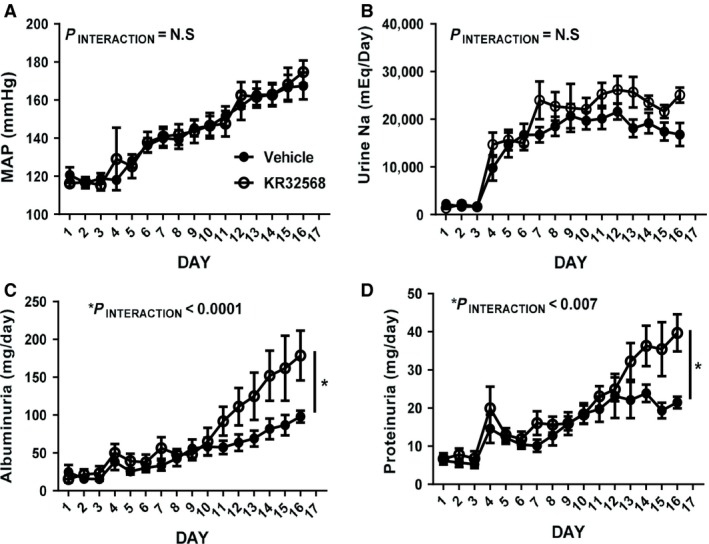
MAP and urinary output in high‐salt‐fed Dahl SS rats. MAP in Dahl SS rats determined by indwelling catheter is shown in panel A. Rats were switched from a 0.4% NaCl containing diet to an 8% NaCl containing diet following Day 3. On Day 6, rats were either maintained on vehicle (closed circles) or vehicle infusions exchanged with 2 mg/kg/day KR32568 (closed circles). *Y*‐axis, average resting MAP between 9 am and 12 pm (mmHg). *X*‐axis, day. B shows 24‐h urinary Na^+^ output. Legend as for panel A except: *Y*‐axis = urinary Na^+^ output *μ*Eq/day. C shows total daily urine albumin excretion. Legend as for panel A except: *Y*‐axis = urinary albumin excretion *μ*g/day. D shows total daily urine albumin excretion. Legend as for panel A except: *Y*‐axis = urinary protein excretion *μ*g/day. Capped bars indicate significant difference between identified groups compared to vehicle using interaction term of two‐way repeated‐measures ANOVA comparing time and MAP responses. Significance *P* < 0.05.

Baseline measurements of blood pressure and urine output were made before switching rats to a high NaCl (8%) salt diet following Day 3. MAP and urine Na^+^ excretion increased immediately (Fig. 3A and B) following high NaCl feeding. Rats were maintained on vehicle infusions for 3 days to allow urinary output to stabilize before replacing infusions with KR32568 (2 mg/kg/day) or maintaining vehicle infusions for the remainder of the protocol (Day 6–17). KR32568 did not altered blood pressure or Na^+^ excretion in high‐NaCl‐fed WT rats (Fig. 3A and B). Renal proteinuria and microalbuminuria which are indicators of glomerular barotrauma (Yoshioka et al. 1988), however, were markedly increased in KR32568‐treated animals compared to vehicle between Day 12 and Day 17 (Fig. 3C and D).

## Discussion

The current study was based on three previous observations. First, clinical trials involving the use of NHE‐1 inhibitors in patients with heart disease were halted due to increased occurrence of thromboembolic stroke, but underlying reasons remained unknown. Second, our previous studies showed that cellular acidification and low intracellular Na^+^ levels promote Hv1‐dependent ROS production in renal tubular cells and peritoneal macrophages. Third, deletion of Hv1, which is found in only in microglia in the brain, reduces ischemic injury in mice by dampening excess ROS formation. Accordingly, the goals of this translational preclinical study were to determine whether (1) NHE‐1 inhibition before or after ischemic insult worsens neurovascular injury and functional outcomes in thromboembolic stroke, (2) activation of proton channel Hv1 contributes to detrimental effects of NHE‐1 inhibition after stroke, and (3) Hv1 contributes to neurovascular injury and poor outcomes in multiple models of stroke. The working hypothesis was that NHE‐1 inhibition augments worsen neurovascular injury after stroke by activating Hv1. Our novel findings show that (1) treatment with NHE‐1 inhibitor prior to ischemic injury or after reperfusion improves stroke outcomes in both WT and Hv1^−/−^ SS rats and (2) proton channel Hv1 contributes to neurovascular injury and poor outcomes after stroke, especially when there is reperfusion.

The most well‐known function of the voltage‐gated proton channel Hv1 is sustaining NADPH oxidase (NOX) activation during the bacterial killing respiratory burst in phagocytic cells through the extrusion of protons and subsequent compensation of charge and pH (DeCoursey 2013). More recently, we have provided evidence that cellular acidification can drive ROS production in renal tubular epithelial cells, and this production of ROS required the presence of Hv1 (Jin et al. 2014; O'Connor et al. 2016). Moreover, in both renal tubular epithelial cells and peritoneal macrophages, Hv1‐dependent ROS production was enhanced by low intracellular Na^+^ or by NHE‐1 inhibition (Jin et al. 2014). These findings led us to speculate that inhibition of NHE‐1 may drive Hv1‐dependent ROS production in the brain in stroke. Such a relationship could underlie the adverse effects of NHE‐1 inhibition observed on incidence of cerebrovascular events in humans. While our data indicate that interactions between Hv1 and NHE‐1 do not worsen stroke outcomes in permanent and transient ischemia models of stroke, our data may shed some light on the seemingly paradoxical results in humans. In the EXPEDITION trial, NHE‐1 inhibition increased the incidence of cerebrovascular events attributed to thromboembolic strokes (Mentzer et al. 2008). Interestingly, while having no effect on Na^+^ excretion or blood pressure, chronic infusion of the NHE‐1 antagonists KR32568 significantly enhanced proteinuria in high‐salt‐fed Dahl salt‐sensitive rats when compared to vehicle (saline) infusion. As this increased proteinuria was only observed in rats following elevations in MAP above ~160 mmHg, these data are strongly indicative of a loss of renal myogenic autoregulatory responses leading to barotrauma at the glomerular capillaries and subsequent protein leak (Yoshioka et al. 1988). Similar dysfunction of myogenic autoregulation in the cerebral circulation could account for increased incidence of stroke with prolonged NHE‐1 inhibition. While preliminary, these data suggest further studies directly investigating the effect of NHE inhibition on cerebral autoregulatory responses or of longer duration in spontaneous models of stroke may be warranted.

Generation of excessive ROS after ischemia contributes to cell death and tissue damage in the brain. In the brain, Hv1 was reported to be functionally expressed only in the microglia, as evidenced by proton currents that were inhibited by zinc, a well‐established antagonist of this channel (Wu et al. 2012). The same study also showed that Hv1^−/−^ mice developed significantly less neuronal injury 1 or 3 days after ischemic stroke induced by permanent and transient MCAO. While this model was a global knock‐out of the channel, additional studies suggested that microglial Hv1 is critical in maintaining NOX2 activity, ROS generation, and tissue injury. A more recent study showed that microglial Hv1 channel‐mediated ROS production not only contributes to oligodendrocyte progenitor cell death after hypoxic injury but also impairs the maturation of the surviving cells (Yu et al. 2018). The current study used Hv1‐deficient rats developed on the Dahl SS rat genetic background and expanded the impact of Hv1 deletion on stroke outcomes by using both permanent and transient ischemia models induced by suture and embolic occlusion of MCA. Moreover, not only neuronal but vascular injury induced by ischemia was assessed by measuring edema and HT. Ischemia reperfusion injury increased vascular injury in WT rats, and both edema and HT were attenuated in Hv1^−/−^ rats providing evidence for the first time that Hv1 deletion confers both neuronal and vascular protection after ischemia in rats.

Given our data indicating that NHE‐1 inhibition can enhance Hv1‐dependent ROS production (Jin et al. 2014) and that chronic NHE‐1 inhibition predisposes to increased mortality as a result of thromboembolic stroke in clinical studies (Mentzer et al. 2008), we asked the question whether pharmacological inhibition of NHE‐1 with KR32568 before the ischemic injury will worsen ischemic stroke by activation of Hv1‐dependent ROS. The current study tested the impact of NHE‐1 inhibition on stroke outcomes using two treatment paradigms in thromboembolic model of stroke. Pretreatment for 3 days before stroke as well as treatment started after the ischemic injury reduced infarct size and secondary bleeding into the brain and improved sensorimotor deficits. The reduction in HT index and brain Hb content was more pronounced in the pretreatment group. While pretreatment period was short, these findings suggest that NHE‐1 inhibition before an ischemic event does not worsen tissue injury caused by thromboembolic stroke. Our results show that NHE inhibition provided neurovascular protection and improved functional outcome, and while it was not statistically significant, this protection was more apparent in animals pretreated with KR32568 versus those receiving KR32568 postischemia. Importantly, and in contrast to our hypothesis, the beneficial effects of both pre‐ and post‐treatment with KR32568 were similar in WT and Hv1^−/−^ rats, indicating that the beneficial effects of NHE inhibition in the poststroke period were not greater in rats lacking Hv1 due to loss of a potentially deleterious increase in Hv1 activation. While we did not measure brain or microglia ROS levels after stroke, our finding that the neurovascular protective effect of Hv1 deletion is greater when there is reperfusion after ischemia strongly suggests a ROS‐dependent mechanism. Interestingly, a recent study showed that microglia from Hv1‐deficient mice generate greater ROS than WT mice (Kawai et al. 2018), suggesting an unconventional regulation of ROS by Hv1 in microglia.

Our results are consistent with previous reports indicating neural protection with NHE‐1 inhibition. Since intracellular acidification is an important factor contributing to cell death after an ischemic event, numerous studies investigated the effect of NHE‐1 inhibition on stroke outcomes. Lam and colleagues reported that neurons use Na^+^/H^+^ exchange as a major mechanism of proton flux to sustain NOX2 activation and genetic deletion of neuronal NHE‐1 prevents superoxide production and cell death in vivo and in vitro (Lam et al. 2013). Another study reported that pharmacological inhibition of NHE‐1 by sabiporide prevented ischemia‐induced increase in blood–brain barrier (BBB) permeability by preventing disruption of tight junction proteins in vivo as well as in an endothelial cell culture model (Park et al. 2010). More recently, it was reported that astrocytic NHE‐1 contributes to neurovascular injury after ischemic stroke and knock‐out of NHE‐1 selectively in astrocytes reduces BBB permeability and reactive astrogliosis after stroke (Begum et al. 2018). However, the models adopted in these studies were with short ischemia time in which the reperfusion was achieved by physically removing the nylon suture, and the NHE‐1 inhibition treatment was given within a narrow time window around the ischemic event. The current study provides additional evidence that NHE‐1 inhibition is protective when given before or after ischemic insult in a clinically relevant embolic model of stroke.

Our studies provide the first data that Hv1 deletion confers both neuronal and vascular protection after ischemia in rats. These data are important as they build on previous reports in mice identifying Hv1 as a potential novel target for the treatment of stroke. In contrast to our hypothesis, inhibition of NHE‐1 with KR32568 provided further protection from ischemic stroke, and the beneficial effects of both pre‐ and post‐treatment with KR32568 were similar in WT and Hv1^−/−^ rats. These data indicate that NHE‐1 inhibition does not promote a potentially deleterious increase in Hv1 activation and ROS production that underlies the increased incidence of cerebrovascular events observed in patients following NHE‐1 inhibition. Since the past preclinical studies by us and others that led to the current study were conducted in male animals, we only used male rats. Further studies in spontaneous models of stroke or studies directly investigating the effect of NHE‐1 inhibition on cerebral autoregulatory responses in both sexes may provide further insight into the pathological mechanisms mediating increased cerebrovascular events following NHE‐1 inhibition in humans.

## Conflict of Interest

None declared.
